# Machine learning models reveal the importance of time-point specific cis-regulatory elements in *Arabidopsis thaliana* wounding response

**DOI:** 10.1093/plcell/koab288

**Published:** 2021-12-07

**Authors:** Sunil Kumar Kenchanmane Raju

**Affiliations:** 1 Assistant Features Editor, The Plant Cell, American Society of Plant Biologists, USA; 2 Department of Plant Biology, Michigan State University, East Lansing, MI, USA

Plant responses to environmental stimuli require complex gene expression reprogramming regulated at multiple levels. Cis-regulatory elements (CREs) can activate or repress gene expression through transcription factor (TF) binding. Moreover, the temporal nature of stress responses adds another layer of complexity wherein sets of genes are regulated differently based on their timing of expression during stress response. Understanding the complex cis-regulatory syntax is essential to predict when and where genes are expressed and how sequence variations lead to downstream changes in gene expression. Recent advances in deep-learning approaches have enabled predictions of gene expression from genomic DNA features (Wang et al., 2020; Meng et al., 2021). Previous studies have established the ability of computational models to predict plant spatial transcriptional response using known and novel CREs (Uygun et al., 2017). However, a global model to uncover regulatory elements affecting temporal responses to environmental stimuli is still lacking.

In *Arabidopsis thaliana* (Arabidopsis), the transcriptional response to wounding has been well characterized by focusing on gene expression changes in early and late time points without directly assessing potential changes in the cis-regulatory landscape leading to expression shifts at different time points. In new work, **Bethany Moore, Yun Sun Lee, and colleagues (****Moore et al., 2022****)** utilize machine learning approaches incorporating information from curated cis-elements, *in silico* predicted cis-elements, DNase hypersensitivity sites (DHSs), and *in vitro* DNA affinity purification sequencing (DAP-seq) data to characterize wound-induced gene regulation in Arabidopsis. The authors used published wounding gene expression dataset (Kilian et al., 2007) and divided genes into 12 clusters based on their direction of response at each time point. Using two machine learning algorithms, the author’s modeled which regulatory sequences were able to classify genes as differentially regulated or not across different time points and response directions. At first, the authors used 52 known CREs associated with wounding, which showed a median F-measure score of 0.68 which is better than random expectation (F-measure score of 0.5). Models trained with incorporation of additional regulatory information such as DAP-seq and DHSs only marginally improved model performance in classifying genes within a cluster.

To understand features that are particularly important for predicting transcriptional response to wounding, the authors ranked top 10 features in each cluster which revealed a known rapid wound response (RWR) regulatory element, CGCGTT, as an early wound response CRE. Interestingly, this RWR element was not the most important contributing feature in predicting gene expression responses during later time points. By contrast, open chromatin sites (DHSs) particularly, at later time points showed up as important features for predicting expression regulation (Figure). To test additional regulatory sequences controlling wounding response, the authors used a *k*-mer finding approach to identify putative cis-regulatory elements (pCREs). Models trained with pCREs alone performed better than models built with known CREs, DAP-seq, and DHSs (F-measure range = 0.73∼0.81), indicating the presence of several previously unknown pCREs. When the authors considered average importance ranks of all features in the model, DHSs turned out to be some of the most highly ranked features, but specific pCREs proved to be more important than DHS features at most time points.

**Figure:**
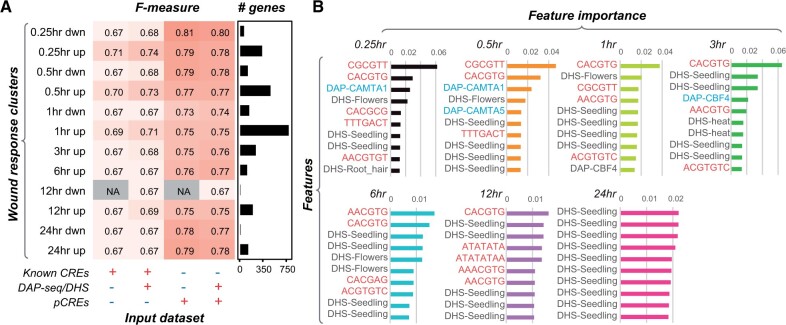
Model performance and feature importance of *A. thaliana* wound response cluster predictions. A, F-measures of Random Forest models trained with different input features. F-measure, which jointly considers precision and recall is used to measure model performance. B, Top 10 features in models built using known CREs (orange), DNA affinity purification-sequencing (cyan) and DNAse I hypersensitivity sites (black) for up-regulated time-point clusters. Adapted from Moore et al. (2022) Figure 2A and B.

Furthermore, the authors experimentally validated the importance of pCREs in early wound response. CRISPR-Cas9 promoter edits in selected genes, *JASMONATE-ZIM-DOMAIN-PROTEIN 2* *(JAZ2*) and *GEM-RELATED 5* *(GER5*) confirmed the role of pCRE “CCGCGT” in wound-responsive expression of *GER5*. Additional *in vitro* binding experiments show other pCREs GTCGGC, GTCACA, and ACACGT can bind to their predicted TF, suggesting their importance in wound response at later time points. Incorporating published jasmonic acid (JA) hormone treatment data with wounding treatments, the authors were able to generate JA-dependent and JA-independent gene subclusters and show that these two responses were regulated by different sets of pCREs and chromatin accessibility patterns. Finally, they also show pCREs important to the cascading response of genes in the glucosinolate biosynthesis from tryptophan pathway across wounding time series, suggesting that CREs acting at different time points regulate pathways triggered by environmental stimuli.

Overall, this work provides a comprehensive model of wound-responsive gene regulation over time and ranks the most important pCREs in predicting transcriptional response to wounding. More importantly, the authors use CRISPR-Cas9 genome editing to precisely modify a predicted binding site to successfully validate its role in Arabidopsis wounding response. The addition of treatment-specific DAP-seq and DHS data could undoubtedly improve model predictions. However, pCREs specific to wound-related pathways such as JA dependent and glucosinolate biosynthesis, and highly ranked features at different time points could be prioritized for experimental validation in future wounding studies.
